# Association between HOMA-IR and ovarian sensitivity index in women with PCOS undergoing ART: A retrospective cohort study

**DOI:** 10.3389/fendo.2023.1117996

**Published:** 2023-03-09

**Authors:** Yan Li, Yiwen Wang, Hai Liu, Shaodi Zhang, Cuilian Zhang

**Affiliations:** ^1^ Reproductive Medicine Center, People’s Hospital of Zhengzhou University, Henan Provincial People’s Hospital, Zhengzhou, Henan, China; ^2^ Department of Gynaecology and Obstetrics, Xinjiang Production and Construction Corps 13 Division Red Star Hospital, Hami, Xinjiang, China

**Keywords:** insulin resistance, ovarian sensitivity, OSI, HOMA-IR, ART

## Abstract

**Introduction:**

Insulin resistance (IR) may play a central role in the pathophysiology of polycystic ovary syndrome (PCOS). Controlled ovarian stimulation (COS) in PCOS women in the setting of assisted reproductive technology (ART) is always a challenge for clinicians. However, it remains unclear whether IR in women with PCOS correlates with reduced ovarian sensitivity to exogenous gonadotropin (Gn). This study aimed to explore the association between homeostasis model assessment of insulin resistance (HOMA-IR) and ovarian sensitivity index (OSI).

**Methods:**

In this retrospective cohort study, we explored the association between Ln HOMA-IR and Ln OSI based on smoothing splines generated by generalized additive model (GAM). Then the correlation between HOMA-IR and OSI was further tested with a multivariable linear regression model and subgroup analysis.

**Results:**

1508 women with PCOS aged 20-39 years undergoing their first oocyte retrieval cycle were included consecutively between 2018 until 2022. We observed a negative association between Ln HOMA-IR and Ln OSI by using smoothing splines. In multivariable linear regression analysis, the inverse association between Ln HOMA-IR and Ln OSI was still found in PCOS women after adjustment for potential confounders (β = -0.18, 95% CI -0.25, -0.11). Compared with patients with the lowest tertile of HOMA-IR, those who had the highest tertile of HOMA-IR had lower OSI values (β = -0.25, 95% CI -0.36, -0.15).

**Discussion:**

Our study provided evidence for the inverse correlation between IR and the ovarian sensitivity during COS in PCOS women. Herein, we proposed new insights for individualized manipulation in PCOS patients with IR undergoing ART.

## Introduction

Polycystic ovary syndrome (PCOS) is a highly prevalent endocrine disorder affecting 6–21% of women of reproductive age (1–3). Oligomenorrhea, obesity, infertility, hyperandrogenemia and insulin resistance (IR) constitute the common features of PCOS (4–6). In PCOS women, IR may play a central role in the pathophysiology, which occur with a prevalence of 77.5% in overweight and 93.9% in obese subjects (7). Homeostatic model assessment (HOMA-IR) is broadly used as a surrogate measure of IR in clinical research (7, 8).

In the setting of assisted reproductive technology (ART), controlled ovarian stimulation (COS) in PCOS women is a challenge for clinicians. The ovarian response to COS is reported to vary widely among PCOS patients. While some patients are more likely to show resistance to stimulation, other PCOS women may experience an exaggerated response (9, 10). Thus, the identification of heterogenous ovarian sensitivity in PCOS populations is the key to striking a balance between ovarian hyperstimulation syndrome (OHSS) and poor ovarian response (POR). Some clinical parameters, including age, body mass index (BMI), anti-mullerian hormone (AMH), and antral follicle count (AFC) have been widely used as predictive markers of ovarian response (11). However, these markers cannot properly reflect the dynamic process of follicular growth in response to exogenous gonadotropin (Gn) (12). Recently, ovarian sensitivity index (OSI) has been suggested as an evaluation of ovarian response to Gn stimulation in ART (12). Higher values of OSI were associated with better ovarian response and greater odds of pregnancy (12–14).

The association between IR and ovarian function in PCOS women has been a debating issue. Previous studies have shown that hyperinsulinemia could promote early folliculogenesis which may result in hyper-response to COS (15, 16). In contrast, researchers observed increased fasting insulin was associated with decreased numbers of large antral follicles in PCOS patients (17). Emerging evidence for the interplay between IR and atresia of antral follicles in PCOS has been described (18, 19). In addition, some researchers reported IR may have an adverse effect on the developmental potential of oocytes when considering the reduced maturation rate (20).

Therefore, it remains unclear whether IR in women with PCOS correlates with reduced ovarian sensitivity to Gn. To our knowledge, few studies have evaluated the association between HOMA-IR and OSI during ART procedures. The aim of this retrospective cohort study was to investigate the association between HOMA-IR and OSI in PCOS women scheduled for *in vitro* fertilization (IVF) or intracytoplasmic sperm injection (ICSI) cycles, which may offer useful guidance to clinicians for individualized infertility therapies.

## Materials and methods

### Patients

This study was a retrospective cohort analysis. Women who underwent a standard Gonadotropin releasing hormone (GnRH) agonist or GnRH antagonist protocol in their first IVF/ICSI treatment cycle at reproductive medicine center of Henan Provincial People’s Hospital between June 2018 and May 2022 were consecutively included. Diagnosis of PCOS was based on the Rotterdam criteria (21). Body mass index (BMI) of ≥ 24 kg/m^2^ was defined as overweight and BMI of ≥ 28 kg/m^2^ was defined as obesity according to Working Group on Obesity in China (22, 23).

We included individuals (BMI ≥ 18.5 kg/m^2^) aged between 20 and 39 years with complete data on IR, including fasting glucose (FG), fasting serum insulin (FINS). The exclusion criteria were as follows: FG > 7 mmol/l, untreated thyroid diseases, subjects had received anti-diabetic medications within 3 months prior to evaluation, preimplantation genetic testing (PGT), canceling oocyte retrieval and oocytes freezing.

### FG and FINS measurement

Basal FSH, LH, estradiol, total testosterone, progesterone and prolactin were done during the 2-4 days of the menstrual cycle. Fasted blood samples were collected to measure biochemical markers, including insulin, glucose and thyroid-stimulating hormone (TSH). The inter-assay laboratory coefficient of variation (CV) of FG testing was lower than 3.5%, which was detected by ADVIA2400ChemistrySystem (ADVIA 2400, SIEMENS, Germany). FINS concentration was determined by the electro-chemiluminescence immunoassay method (CV < 3.2%) on the full-automatic chemiluminescence immunoassay analyzer (Cobas8000 e602; Roche Diagnostics GmbH, Mannheim, Germany) in the laboratory of the Department of Reproductive Endocrinology at Henan Provincial People’s Hospital. Our laboratory is checked for qualification by the External Quality Assessment of Clinical Laboratory Center annually (Ministry of Health of the People’s Republic of China, Beijing, China).

### Indicator calculation

HOMA-IR and OSI were assessed by formula as follows: HOMA-IR = FBG (mmol/L) x FINS (μU/ml)/22.5 (24); OSI = [(Number of retrieved oocytes/Total gonadotropin dose) × 1,000] (12); BMI was calculated according to the formula, weight (kg)/height (m)^2^. Implantation rate was defined as the number of gestational sacs divided by the number of transferred embryos. Clinical pregnancy rate was calculated by the ratio of clinical pregnancy cycle to the total embryo transfer (ET) cycle. Early miscarriage was referred to intrauterine pregnancy loss before 12 weeks of pregnancy, while late miscarriage was defined as a pregnancy loss prior to 28 weeks of gestational age.

### Controlled ovarian stimulation protocol

COS protocols consisted of GnRH agonist down regulation protocol and GnRH antagonist protocol. These dose step-up regimens were individualized according to women’s age, BMI and ovarian reserve. In GnRH agonist down regulation protocol, subcutaneously injected 0.1 mg triptorelin was scheduled for patients from the 6th-8th day after ovulation to the 18th-22th day until sufficient downregulation of the pituitary was achieved. After that, exogenous Gn and 0.05 mg triptorelin was administered simultaneously until the day of human chorionic gonadotropin (HCG) triggering. In the long-acting GnRH agonist down regulation protocol, patients received a single dose of triptorelin acetate (Diphereline; 3.75mg) on day 2-4 of the menstrual cycle. If downregulation of the pituitary was satisfactory after 30-35 days, exogenous Gn was injected to initiate the cycle. In the GnRH antagonist protocol, Gn was administrated on the 2-3 days of the menstrual cycle, and GnRH antagonist (Cetrotide; 0.25 mg) was added daily from day six to seven of stimulation until the day of HCG triggering. The hCG was administered when at least two follicles had reached a mean diameter of 17-18 mm and the serum estradiol (E2) levels were consistent with the ultrasound findings. Ultrasound-guided follicular aspiration was performed at 35-36 hours after the administration of the hCG injection. High-quality embryos meant day 3 embryos that reached 6 to 8 cell stages with cytoplasmic fragmentation less than 10% and equal size blastomeres.

### Statistical analysis

Owing to skewed distribution, HOMA-IR and OSI values were log e transformed to Ln HOMA-IR and Ln OSI. Continuous variables with normal distribution were expressed as mean ± standard deviation (SD). Continuous variables with skewed distribution were presented median with interquartile range (IQR). Categorical variables were expressed as frequency (percentage). The differences between HOMA-IR tertiles were compared using the one-way analysis of variance (normal distribution), Kruskal-Wallis test (skewed distribution) for continuous variables and Pearson’s chi-squared test, or Fisher’s exact test for the categorical variables. Multiple comparison posttest was conducted by using the Bonferroni correction.

The association of Ln HOMA-IR with Ln OSI was fitted and presented as smoothing splines which was generated by a generalized additive model (GAM) after adjustments for age, BMI, AFC, AMH, the initial Gn dose, basal FSH and COS protocol.

To analyze whether Ln HOMA-IR was independently associated with Ln OSI, multivariable linear regression models were used. These models included crude model (not adjusted for covariates), model 1 (adjusted for age, BMI, AMH and AFC), model 2 (adjusted for age, BMI, AMH, basal FSH, initial Gn dose, AFC, and COS protocol). As sensitivity analysis, HOMA-IR was then divided into tertiles and treated as a categorical variable, with the lowest tertile used as the reference. In addition, we performed linear trend tests to obtain *P* for trend by entering the median value of each HOMA-IR category as a continuous variable in the models.

Subgroup analysis was performed for examination of the association of Ln HOMA-IR with Ln OSI in the strata of age, BMI, AMH, initial Gn dose, and COS protocol. Next, we use log likelihood ratio test to obtain a *P*-value for interaction for examining the statistical significance of the difference in each subgroup.

Statistical analysis was undertaken by using software packages R (http://www.R-project.org, The R Foundation) and Empower (R) (www.empowerstats.com; X&Y Solutions, Inc., Boston, MA). A two-tailed *P* value < 0.05 was considered statistically significant.

## Results

### Patient disposition

**Figure 1:**
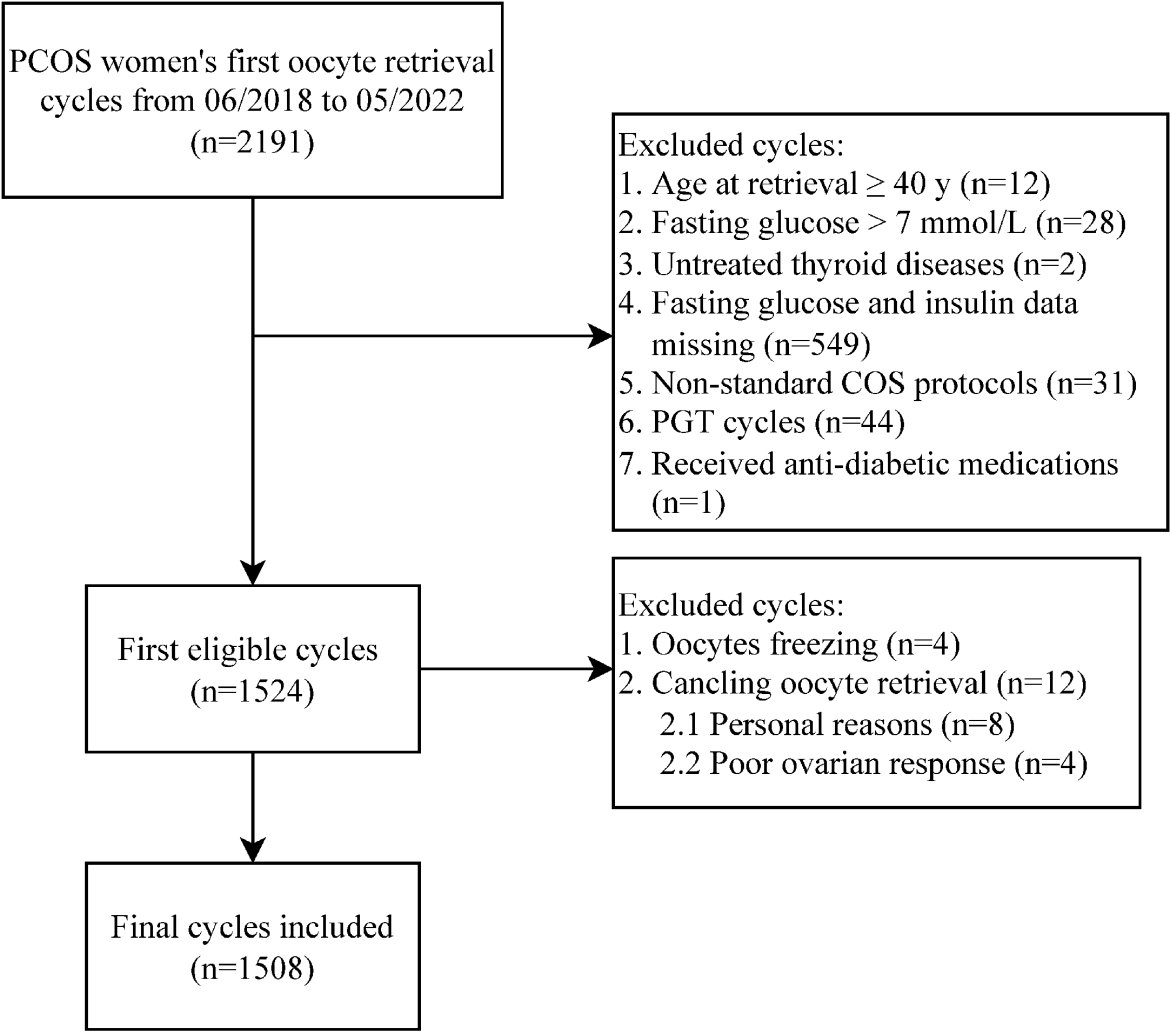
Flowchart of data collection process. PCOS, polycystic ovary syndrome; COS, controlled ovarian stimulation; PGT, preimplantation genetic testing.

Data from women having PCOS undergoing their first oocyte retrieval cycles were analysed. A total of 2191 medical records between June 2018 and May 2022 were screened and 1508 IVF/ICSI cycles were finally included in the analysis (**Figure 1**).

### The clinical parameters of patients

Patient characteristics were presented in **Table 1**. Subjects with higher HOMA-IR tended to be younger and had higher levels of BMI, AFC, FINS, FG, the initial Gn dose, total Gn dose and duration of Gn used. The levels of AMH, basal FSH, basal LH, dominant follicle count on trigger day, number of retrieved oocytes, metaphase II (MII) oocytes, embryos and OSI values were prone to be decreased across the HOMA-IR tertiles. With regards to the clinical outcomes, as shown in **Supplementary Figure S1**, the early miscarriage rate was significantly higher in T3 group when compared with that of T1 subjects (*P* < 0.05). No significant difference was detected for implantation rate, clinical pregnancy rate and late miscarriage rate (*P* > 0.05).

**Table 1:** The clinical parameters of the study population.

Variables	Groups of cycles according to the tertiles of HOMA-IR			*P* value^1^
	T1 (< 2.32)	T2 (2.32 - 3.87)	T3 (> 3.87)	
Number	503	502	503	
Age (y)	29.44 ± 3.55	28.87 ± 3.53	28.59 ± 3.82	<0.001
BMI (kg/m^2^)	22.77 ± 2.76	25.03 ± 3.38	27.95 ± 3.56	<0.001
AMH (ng/ml)	7.61 (5.29-10.89)	7.30 (5.07-10.44)	6.75 (4.31-9.65)	<0.001
Basal FSH (mIU/ml)	5.93 ± 1.41	5.76 ± 1.50	5.48 ± 1.43	<0.001
Basal LH (mIU/ml)	8.44 (5.66-13.38)	7.75 (5.02-11.92)	7.29 (4.50-10.54)	<0.001
AFC	22.75 ± 3.72	23.17 ± 3.54	23.97 ± 3.86	<0.001
FG (mmol/l)	4.63 ± 0.47	4.84 ± 0.48	5.07 ± 0.54	<0.001
FINS (μU/ml)	8.05 ± 2.15	14.17 ± 2.40	27.59 ± 10.67	<0.001
HOMAIR	1.72 (1.37-1.99)	3.00 (2.61-3.41)	5.38 (4.50-7.00)	<0.001
COS protocol				0.034
GnRH agonist	406 (80.72%)	427 (85.06%)	435 (86.48%)	
GnRH antagonist	97 (19.28%)	75 (14.94%)	68 (13.52%)	
Initial Gn dose (IU)	128.88 ± 31.38	135.21 ± 28.54	147.69 ± 36.24	<0.001
Total Gn dose (IU)	1826.78 ± 875.73	2183.34 ± 1168.43	2730.55 ± 1218.45	<0.001
Duration of Gn (d)	11.22 ± 3.10	12.07 ± 3.56	13.15 ± 3.51	<0.001
Dominant follicle count on trigger day	9.87 ± 4.68	9.54 ± 4.84	9.09 ± 4.49	0.028
Retrieved oocytes	14.00 (10.00-20.00)	13.00 (8.00-18.00)	12.00 (8.00-17.50)	<0.001
MII oocytes	12.00 (8.00-16.00)	11.00 (7.00-16.00)	10.00 (7.00-15.00)	<0.001
Embryos count	7.00 (4.00-10.00)	6.00 (3.00-10.00)	5.00 (3.00-9.00)	0.003
Endometrium on ET day (mm)	10.82 ± 2.45	11.06 ± 2.42	11.00 ± 2.72	0.619
OSI	8.37 (5.14-13.33)	6.40 (3.88-10.79)	4.57 (2.85-7.23)	<0.001

BMI, Body Mass Index; AMH, anti-mullerian hormone; FSH, follicle stimulating hormone; LH, luteinizing hormone; AFC, antral follicle count; HOMA-IR, homeostatic model assessment of insulin resistance; FG, fasting glucose; FINS, fasting serum insulin; COS, controlled ovarian stimulation; GnRH, gonadotropin releasing hormone; Gn, gonadotropin; OSI, ovarian sensitivity index; MII, metaphase II; ET, embryo transfer; A two-tailed P value < 0.05 was considered statistically significant.

### Associations between HOMA-IR and OSI

**Figure 2:**
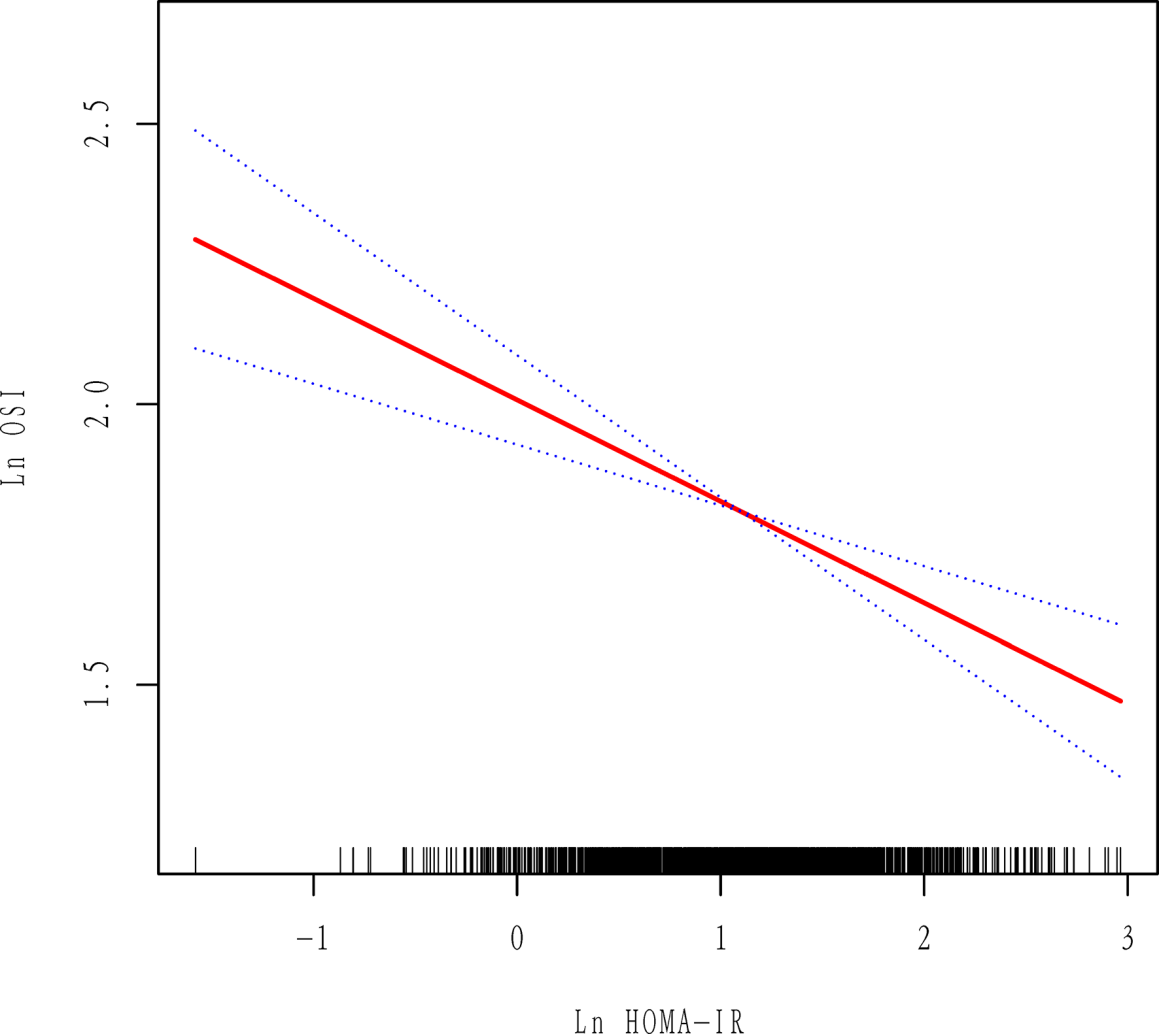
Association between Ln HOMA-IR and Ln OSI. All adjusted for age, BMI, AMH, AFC, initial Gn dose, basal FSH and COS protocol. Red line represents the smooth curve fit between variables and the blue dotted curves represents the 95% of confidence interval.

The data distribution of HOMA-IR and OSI was strongly skewed. Thus, we performed log e transformation (Ln HOMA-IR and Ln OSI) before analysis. First, we observed a negative association between Ln HOMA-IR and Ln OSI after adjustment for potential confounders by using smoothing spline fitting curves in GAM (**Figure 2**). Then, a multivariable linear regression model was performed to analyze the association of Ln HOMA-IR with Ln OSI levels. In addition, we performed sensitivity analysis where HOMA-IR was divided into three groups based on tertiles.

As displayed in **Table 2**, univariable linear regression analysis showed that, the level of Ln HOMA-IR was negatively associated with Ln OSI values (β = -0.39, 95% CI -0.45, -0.32). After adjustment for potential confounders, the negative association between Ln HOMA-IR and Ln OSI was still found, as shown in model 1 (β = -0.14, 95% CI -0.21, -0.07) and model 2 (β = -0.18, 95% CI -0.25, -0.11) by multivariable linear regression analysis. As sensitivity analysis, HOMA-IR was then divided into tertiles and treated as a categorical variable, with the lowest tertile used as the reference. A graded negative association was discovered across the groups (*P* for trend < 0.001). Compared with participants who had HOMA-IR in T1 (HOMA-IR < 2.32), those with HOMA-IR in T2 (HOMA-IR 2.32 - 3.87) and T3 (HOMA-IR > 3.87) had lower Ln OSI (β = -0.25, 95% CI -0.34, -0.15 and β = -0.57, 95% CI -0.67, -0.48, respectively). After adjusting for the potential confounders, the Ln OSI remained consistently lower in T3 compared with T1 in model 1 (β = -0.21, 95% CI -0.31, -0.10). In model 2, the Ln OSI remained significantly lower in T2 and T3 groups when compared with T1 group (β = -0.10, 95% CI -0.19, -0.00 and β = -0.25, 95% CI -0.36, -0.15, respectively).

**Table 2:** Multivariate linear regression for association of Ln HOMA-IR with Ln OSI.

Variable	Crude Model		Model 1		Model 2	
	β (95%CI)	*P* value	β (95%CI)	*P* value	β (95%CI)	*P* value
Ln HOMA-IR	-0.39 (-0.45, -0.32)	<0.0001	-0.14 (-0.21, -0.07)	<0.0001	-0.18 (-0.25, -0.11)	<0.0001
HOMA-IR tertile						
T1 (< 2.32)	0 (reference)		0 (reference)		0 (reference)	
T 2 (2.32 - 3.87)	-0.25 (-0.34, -0.15)	<0.0001	-0.07 (-0.17, 0.02)	0.1164	-0.10 (-0.19, -0.00)	0.0391
T 3 (> 3.87)	-0.57 (-0.67, -0.48)	<0.0001	-0.21 (-0.31, -0.10)	0.0002	-0.25 (-0.36, -0.15)	<0.0001
*P* for trend		<0.001		<0.001		<0.001

HOMA-IR, homeostatic model assessment of insulin resistance; Crude model adjust for none; Model 1 adjust for: age, BMI, AMH, and AFC; Model 2 adjusted for: age, BMI, AMH, basal FSH, initial Gn dose, AFC, and COS protocol.

### Subgroup analysis and effect modification

As presented in **Table 3**, subgroup analysis was performed to explore whether the other variables, including age, BMI, stratification of AMH, initial Gn dose and COH protocol, might influence the association between HOMA-IR and OSI. The subgroups of age, AMH and BMI were stratified according to the clinical cutoff point. The subgroup analysis revealed the inverse association between Ln HOMA-IR and Ln OSI was consistent and significant in the following subgroups: BMI, AMH and initial Gn dose. None of the abovementioned variables significantly modified the association between HOMA-IR and OSI (*P* for interaction > 0.05 for all covariates). In the subgroups of patients with age ≥ 35 y and using GnRH antagonist protocol, the inverse associations of Ln HOMA-IR with Ln OSI was not statistically significant although the regression coefficient (β) was negative.

**Table 3:** Subgroup analysis of the association between Ln HOMA-IR and Ln OSI.

Subgroups	Subjects n (%)	β (95%CI)	*P* value	*P* for interaction
Age tertile (y):				0.5570
< 35	1415 (93.83%)	-0.18 (-0.26, -0.11)	<0.0001	
≥ 35	93 (6.17%)	-0.11 (-0.45, 0.23)	0.5300	
BMI categories:				0.3930
Normal weight	628 (41.64%)	-0.19 (-0.29, -0.09)	0.0002	
Overweight/obese	880 (58.36%)	-0.25 (-0.35, -0.16)	<0.0001	
AMH (ng/ml):				0.2802
≤ 5	382 (26.45%)	-0.14 (-0.27, -0.01)	0.0378	
> 5	1062 (73.55%)	-0.21 (-0.30, -0.12)	<0.0001	
Initial Gn dose (IU):				0.9251
< 150	891 (59.08%)	-0.18 (-0.27, -0.08)	0.0002	
≥ 150	617 (40.92%)	-0.16 (-0.28, -0.04)	0.0089	
COS procotol				0.9834
GnRH agonist	1268 (84.08%)	-0.18 (-0.26, -0.10)	<0.0001	
GnRH antagonist	240 (15.92%)	-0.16 (-0.35, 0.03)	0.1031	

HOMA-IR, homeostatic model assessment of insulin resistance; BMI, Body Mass Index; AMH, anti-mullerian hormone; COS, controlled ovarian stimulation; Gn, gonadotropin; GnRH, gonadotropin releasing hormone. Adjusted, if not stratified, for age, BMI, AMH, basal FSH, initial Gn dose, AFC, and COS protocol.

## Discussion

In this study, we explored the association of HOMA-IR with OSI in a relatively large cohort of women with PCOS. Few studies have been performed on the association between HOMA-IR and OSI. To our knowledge, only one research depicted a decreased OSI in 131 IR-PCOS women compared with 52 non-IR PCOS subjects (11). However, the sample size was small and the effect size of IR on OSI was unclear. The findings of this study showed that HOMA-IR inversely and consistently correlated with OSI in PCOS patients undergoing ART after adjusting for potential confounders. The present study indicated that IR may be associated with reduced ovarian sensitivity to exogenous Gn during COS.

PCOS has been suggested to possess heterogeneous subpopulations, including lean PCOS, overweight/obese PCOS and PCOS women with serum AMH > 5 ng/ml (25, 26). A high level of AMH (> 5 ng/ml) has been reported to be correlated with ovarian hyper-response (27, 28). Interestingly, our results indicated that the negative association of HOMA-IR with OSI remained consistent in BMI and AMH subgroups, suggesting the inverse association between HOMA-IR and OSI was independent. Thus, we assumed that the negative association between HOMA-IR and OSI may be intrinsic to PCOS, which should be managed early on.

Mechanisms underlying decreased ovarian sensitivity in PCOS women with IR have not yet been determined. Johnstone et al. proposed a role for insulin in suppressing growth of 5-10mm follicles in the follicular phase which may contribute to anovulation in PCOS (17). Women with PCOS exhibited diminished initial E2 responses to FSH compared with controls (29). Evidence from IR mouse models indicated that maternal IR contributed oxidative stress and defective mitochondrial function in germinal vesicle (GV) and metaphase II (MII) oocytes, which potentially impaired oocyte quality (30). Although it is thought that genetic variations of FSH receptors influence the degree of ovarian response to stimulation (31). However, studies performed on this subject showed contradictory results. A difference in response for specific FSH receptor subtypes may be very small, and not likely to be the basis for the wide variation in the number of oocytes retrieved in response to COS (32, 33). In light of these observations, IR may be linked to ovarian dysfunction in PCOS.

IR plays a key role in the multisystem pathophysiology of PCOS. The reported prevalence of IR in women with PCOS has ranged from about 12% to over 60% due to the use of different cutoffs, different tests, and different populations (34). HOMA-IR is a measurement frequently used in clinical studies, but no established cutoffs exist (34, 35). Researchers have used different methods to describe cutoff values such as the 66th percentile, the top quartile, and the 90th or 95th percentile (35–37). Till now, IR have been reported to occur at HOMA-IR levels that range from 2.1 to 3.8 (36, 38–40). Many studies also selected HOMA-IR of 2.5 as an indicator of IR based on the original study by Matthews et al. (24). In our study, similar to the measurements of IR abovementioned, our results indicated that PCOS women who had HOMA-IR values of tertile 2 (2.32 - 3.87) and tertile 3 (> 3.87) had significant decreases in OSI values. Besides, the negative association between HOMA-IR and OSI remained consistent in multivariable linear regression analysis, which suggested IR may be associated with decreased ovarian sensitivity.

ART for PCOS patients is always challenging due to the exaggerated or suboptimal ovarian response to Gn. The current results indicated that PCOS women with high HOMA-IR values entailed an extended stimulation phase and a higher number of Gn ampules, which could lead to a decreased OSI. OSI is a better representation of ovarian response rather than the number of oocytes retrieved and the total Gn dose (41). Moreover, the subgroup analysis showed the negative association of HOMA-IR with OSI remained consistent in stratification of initial Gn dose. A low-dose Gn stimulation strategy for PCOS patients was recommended (16). As previously reported, it was suggested that a low starting Gn dose of < 150 IU/day and 25-IU incremental doses every third day should be considered in a COS protocol for PCOS patients with a high HOMA-IR score (42). It is necessary to decide on both the initial Gn dose and the incremental dose when a low-dose step-up regimen is used. The initial Gn dose was usually calculated depending on age, BMI and ovarian reserve. In this regard, by taking into account these parameters as well as HOMA-IR score, and hence adjusting the starting and incremental Gn dose appropriately, the value of OSI may be increased and the ART outcome may be improved.

In addition, we found the early miscarriage rate was significantly increased in the group of patients with high HOMA-IR values. No significant differences were obtained in implantation rate, clinical pregnancy rate and late miscarriage rate. The findings were in line with the previous studies, which indicated the adverse effect of IR on reproduction (43, 44).

Some limitations existed in our study. First, it was not designed as a prospective study. Chinese ethnicity of our participants may limit generalization of the findings to different ethnic groups. Patient diagnosis, age, and COS protocols may vary from study to study. Moreover, the sample size in PCOS subgroups with age > 35y and using GnRH antagonist protocol was small. As reported, it is advantageous to consider ART outcomes from all cycles in order to draw clinically relevant inferences and to maximize study power (45). In this study, we restricted analysis to the first cycle because of the concern for potential bias, such as weight loss or anti-hyperinsulinemia medications.

## Conclusion

In conclusion, this study, carried out in a cohort of PCOS women undergoing ART, demonstrated that HOMA-IR value was negatively associated with OSI. By taking into account the insulin resistant status, it may help clinicians for individualized ovarian stimulation in PCOS patients. Future research will be needed to validate our results and investigate the mechanistic links between IR and ovarian sensitivity.

## Data availability statement

The original contributions presented in the study are included in the article/**Supplementary Material**, further inquiries can be directed to the corresponding author.

## Ethics statement

The study was approved by the Ethics Committee of the Henan Provincial People’s Hospital (No. 2022139). The study was conducted in accordance with the Helsinki Declaration and patients’ records were anonymized prior to analysis. The need for individual consent was waived by the committee due to the retrospective character of the study.

## Author contributions

YL and YW conceived the study. HL critically reviewed the study and helped to draft the manuscript; YL wrote the manuscript. SZ and CZ participated in its design and coordination. All authors contributed to the article and approved the submitted version.

## Supplementary material

Supplementary Figure 1 The clinical outcomes of patients with different HOMA-IR values. **P* < 0.05).
