# Supplementary figures and images for: Association between HOMA-IR and ovarian sensitivity index in women with PCOS undergoing ART: A retrospective cohort study
Source: Front Endocrinol (Lausanne). 2023 Mar 9;14:1117996. doi: 10.3389/fendo.2023.1117996 (PMC10034104; doi:10.3389/fendo.2023.1117996)

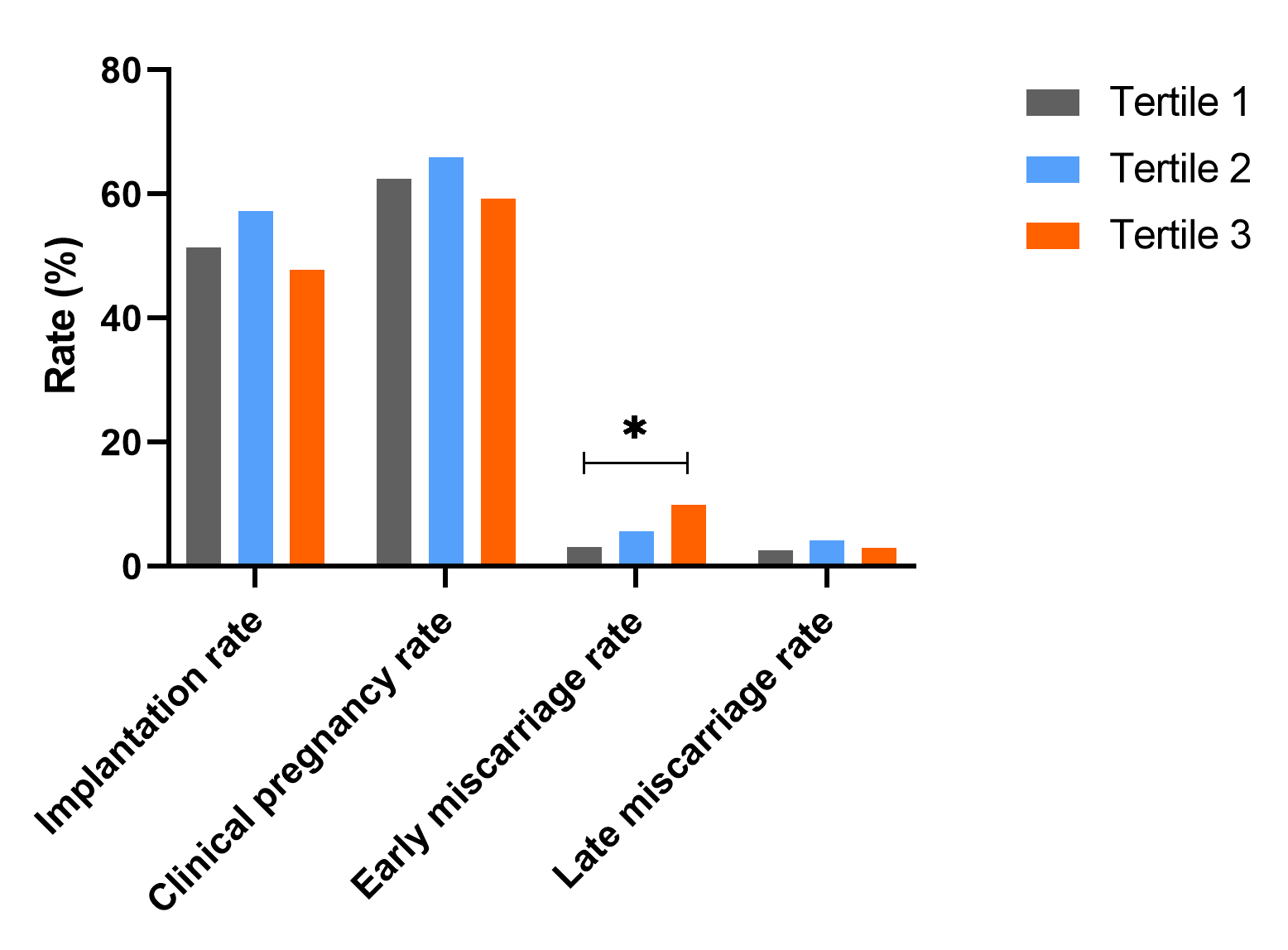

Supplement: Supplementary Figure 1 — The clinical outcomes of patients with different HOMA-IR values. *P < 0.05). [file Image_1.tif]
